# Supplementary material for: MicroRNA-Based Fingerprinting of Cervical Lesions and Cancer
Source: J Clin Med. 2020 Nov 15;9(11):3668. doi: 10.3390/jcm9113668 (PMC7698009; doi:10.3390/jcm9113668)
Supplement: Supplementary file 1 [file jcm-09-03668-s001.pdf]

Supplementary Table S1. Data collected to complete Venn diagram (Figure 1.).

	Group	Total	microRNA species
A	I II III IV	1	miR-21
	I III IV	1	miR-155
	I II	3	miR-15b, miR-210, miR-224
	I III	1	miR-944
	III IV	1	miR-499a
	I	10	miR-7, miR-16, miR-17, miR-25, miR-92a, miR-93, miR-106b, miR-186, miR-378, miR-629
	II	1	miR-27a
	III	3	miR-20b, miR-133b, miR-205
	IV	1	miR-81a
B	I II III IV	1	miR-375
	I II III	1	miR-34a
	I II	1	miR-27a
	I III	1	miR-23b
	III IV	5	miR-125, miR-214, miR-449b, miR-497, miR-664
	I	10	miR-22, miR-23a, miR-24, miR-27b, miR-29, miR-100, miR-125b, miR-143, miR-196b, miR-203
	II	4	miR-145, miR-194, miR-199b, miR-215
	III	3	miR-195, miR-218, miR-223
